# Targeting Mitochondrial Dysfunction in Acute Organophosphate Toxicity: A Preliminary Evaluation of Cyclosporine A

**DOI:** 10.1007/s13181-026-01144-6

**Published:** 2026-08-19

**Authors:** David H. Jang, Jarelis Cabrera, Jonathan Starr, Abigail Insana, Angela N. Viaene, John C. Greenwood, Frances S. Shofer, Johannes K. Ehinger, Shih-Han Kao, Todd J. Kilbaugh

**Affiliations:** 1 Department of Emergency Medicine, Perelman School of Medicine, University of Pennsylvania, 3615 Civic Center Blvd, Philadelphia, PA USA; 2 The Resuscitation Science Center and, Department of Anesthesiology and Critical Care Medicine, Children’s Hospital of Philadelphia, Philadelphia, PA USA; 3 Drexel University, Philadelphia, PA USA; 4 Mitochondrial Medicine, Department of Clinical Sciences Lund, Lund University, Lund, Sweden

**Keywords:** Organophosphate poisoning, Diisopropyl fluorophosphate (DFP), Mitochondrial dysfunction, High-resolution respirometry, Cyclosporine A, Mitochondrial permeability transition pore (mPTP)

## Abstract

**Background:**

Organophosphate (OP) compounds are widely used agricultural chemicals that pose significant public health and chemical threat concerns due to their neurotoxic effects. Although OP toxicity has classically been attributed to acetylcholinesterase inhibition and cholinergic crisis, emerging evidence suggests mitochondrial dysfunction may represent an additional and clinically relevant mechanism of injury. Experimental OP exposure has been associated with impaired electron transport chain activity, reduced ATP production, increased oxidative stress, and disruption of mitochondrial membrane integrity. However, there are currently no established mitochondrial-targeted therapies for OP poisoning. Diisopropyl fluorophosphate (DFP), a well-established OP surrogate, reproduces several neurologic and mitochondrial features of nerve agent exposure. Cyclosporine A (CsA), an inhibitor of the mitochondrial permeability transition pore (mPTP), may represent a potential mitochondrial-targeted intervention.

**Methods:**

This preliminary translational study employed both in vitro and in vivo models to evaluate mitochondrial respiratory dysfunction following acute DFP exposure and to explore the feasibility of mitochondrial-targeted treatment paradigms using CsA. Human donor-derived peripheral blood mononuclear cells (PBMCs) were used for DFP and CsA dose-finding and mitochondrial respiratory characterization using high-resolution respirometry (Oroboros O2k) with standardized substrate–uncoupler–inhibitor titration (SUIT) protocols. In parallel, a non-survivor Sprague–Dawley rat model of acute DFP exposure was developed incorporating invasive hemodynamic monitoring, controlled ventilation, venous blood gas analysis, and CsA treatment paradigms administered either following DFP exposure or as pretreatment prior to exposure. Brain cortical homogenates were prepared for ex vivo respiratory assessment.

**Results:**

Preliminary findings demonstrated that DFP exposure was descriptively associated with impaired mitochondrial respiration across multiple oxidative phosphorylation (OXPHOS)- and electron transport system (ETS)-linked respiratory states in both PBMC and rodent models. DFP exposure was additionally associated with physiologic and metabolic derangements, including acidosis and elevated lactate concentrations. Post-DFP CsA administration was associated with partial preservation of mitochondrial respiration across several respiratory states, while the Pre-DFP CsA group demonstrated respiratory profiles more closely approximating control values in select OXPHOS- and ETS-linked states, accompanied by relatively preserved physiologic and metabolic parameters.

**Conclusions:**

These preliminary findings support the feasibility of integrating mitochondrial respiratory phenotyping into translational models of acute OP toxicity and further support investigation of mitochondrial-targeted strategies in OP-associated mitochondrial dysfunction. The observed respiratory and physiologic trends associated with CsA exposure, particularly in the pretreatment paradigm, provide an exploratory foundation for future preclinical studies evaluating mitochondrial-directed countermeasures in OP poisoning.

## Introduction

Organophosphate (OP) compounds are widely used in agriculture as pesticides and insecticides and are also found in certain chemical warfare nerve agents [1, 2]. Exposure can occur through a variety of means such as occupational contact, environmental contamination, or intentional chemical exposure scenarios [3]. OP poisoning remains a significant public health concern due to its rapid onset of cholinergic toxicity and lack of universally effective medical countermeasures.

The primary mechanism of OP poisoning is the inhibition of acetylcholinesterase (AChE), an enzyme responsible for degrading the neurotransmitter acetylcholine (ACh) in synaptic clefts [4]. This inhibition leads to an accumulation of ACh at the neuromuscular junctions resulting in overstimulation of cholinergic receptors. Clinically, this manifests as a “cholinergic toxidrome” that includes bradycardia, seizures, muscle fasciculations and in severe cases, respiratory failure and death. Beyond cholinergic toxicity, emerging experimental evidence suggests that OP exposure may contribute to mitochondrial dysfunction, including impaired electron transport chain activity, reduced ATP production, increased oxidative stress, and disruption of mitochondrial integrity [5, 6]. Accordingly, mitochondrial dysfunction may represent a complementary target for biomarker development and exploratory therapeutic investigation.

To study OP toxicity under standard laboratory conditions, surrogate compounds such as diisopropyl fluorophosphate (DFP) are commonly used. DFP reproduces key features of nerve agent exposure, including cholinergic crisis, seizure activity, neuroinflammation, and mitochondrial dysfunction [7, 8]. Unlike restricted nerve agents, DFP is commercially available and safe to handle in standard research laboratories, making it a practical model for in vitro and in vivo studies of OP pathophysiology and countermeasure testing. Because DFP exposure reproduces features of both cholinergic toxicity and mitochondrial dysfunction, it provides a practical platform to evaluate candidate mitochondrial-targeted countermeasures. Cyclosporine A (CsA; Sandimmune, Novartis, Basel, Switzerland) is a cyclic peptide best known as an immunosuppressant, but it also inhibits the mitochondrial permeability transition pore (mPTP), a key mediator of mitochondrial injury under oxidative and toxic stress [9, 10]. CsA has demonstrated associations with preservation of mitochondrial function in preclinical models of acute injury, including traumatic brain injury and ischemia–reperfusion, and therefore represents a candidate for exploratory investigation in OP-associated mitochondrial dysfunction [10]. In this preliminary translational feasibility study, we evaluated mitochondrial respiratory changes following DFP exposure and explored CsA-associated mitochondrial respiratory effects using complementary human cell-based and rodent models.

## Material and Methods

### Overall Study Design

This preliminary, proof-of-concept study utilized combined in vivo and in vitro approaches to investigate mitochondrial dysfunction in acute organophosphate toxicity. Human peripheral blood mononuclear cells (PBMCs) isolated from healthy donors were initially used for preliminary dose-finding and feasibility experiments involving both diisopropyl fluorophosphate (DFP) and cyclosporine A (CsA). These PBMC experiments were subsequently used to guide dosing strategies in a non-survivor rodent model of acute DFP poisoning. Independent PBMC experiments were performed for the Control, DFP, and CsA conditions (n = 5–6 replicates per condition).

An equal number of female and male Sprague–Dawley rats (Charles River Laboratories, Wilmington, MA, USA) weighing approximately 200 g were included in the rodent experiments. Due to the exploratory nature of this preliminary study, the rodent sample size was limited (n = 2 per group for Control, DFP, and Post-DFP CsA groups; n = 3 for the Pre-DFP CsA group), limiting formal statistical inference. All rodents were placed in an induction chamber with 5% isoflurane exposure for 1–2 min and, upon induction, positioned on a SurgiSuite Multi-Functional Surgical Platform (Kent Scientific, Torrington, CT, USA) equipped with warming capability and rectal temperature monitoring to maintain a core temperature of 37–38 °C throughout the experiment [11].

Animals were assigned to one of four experimental groups: (1) Control (n = 2), which received no intervention; (2) DFP (n = 2), which received DFP exposure alone; (3) Post-DFP CsA (n = 2), which received CsA administration following DFP exposure during the experimental period; and (4) Pre-DFP CsA (n = 3), which received CsA pretreatment one hour prior to DFP exposure. All procedures were approved by the Institutional Animal Care and Use Committee at the Children’s Hospital of Philadelphia and performed in accordance with the National Institutes of Health Guide for the Care and Use of Laboratory Animals. Human PBMC collection procedures were approved by the University of Pennsylvania Institutional Review Board.

### Perioperative Procedures and Monitoring

After induction, all subjects were placed on the surgical platform, and a rodent cone was used (2–3% isoflurane) to maintain general anesthesia (tail and toe pinch used to assess depth of anesthesia). Isoflurane was weaned to approximately 1–1.5% during DFP/CsA exposure to reduce anesthetic exposure while maintaining adequate anesthesia and minimizing potential physiologic confounding. Immediately upon verification of anesthesia, all animals underwent tracheostomy and were placed on a small animal ventilator (VentElite Small Animal Ventilator,Harvard Apparatus, Holliston, MA, USA). Ventilator settings were respiratory rate 50–55 breaths/min and a pressure of 8–10 cmH₂O to maintain end-tidal CO₂ of 35–45 mmHg.

The right carotid artery was isolated from the internal jugular vein and vagal nerve under 10 × magnification. A Millar SPR-869 Rat PV Catheter (2F, 4E, 6 mm, 15 cm, PI) was advanced into the left ventricle for continuous pressure–volume loop measurements, including cardiac output, stroke volume, and dP/dt. Left femoral vein access was obtained with a 24 g catheter for DFP injection and CsA infusion. All data were recorded with PowerLab 16/35 LabChart 8 Pro software from ADInstruments (ADInstruments, Sydney, Australia).

### Diisopropylfluorophosphate

A wide range of DFP concentrations was initially tested in PBMCs to identify a dose that achieves approximately 50% inhibition of mitochondrial respiration without compromising cell viability. Based on these preliminary in vitro experiments, a final working concentration of 5 mM DFP was selected. The 5 mM DFP working stock was prepared fresh on the day of the experiment by serial dilution of the original 5.76 M DFP stock (Sigma #D0879) in 100% DMSO to minimize injection volume. DMSO vehicle control was tested separately and did not affect mitochondrial function. For each 2.2 mL Oroboros chamber containing approximately 1.5 million PBMCs suspended in MiR05 respiration medium, 1.47 µL of the 5 mM DFP stock was injected to achieve the target final concentration [7, 8]. For rodent experiments, DFP was prepared according to prior publications and administered as a single intravenous bolus (3 mg/kg) over a defined one-hour protocol, with euthanasia at the end of exposure. For a 200 g rat (target dose: 0.6 mg), a 1:100 dilution was prepared by mixing 10 μL of stock with 990 μL sterile saline, and 56.5 μL was injected intravenously [7, 8].

### Cyclosporine A

To evaluate the mitochondrial preservation potential of cyclosporine A (CsA) following DFP-induced mitochondrial dysfunction, a previously tested CsA dose–response curve was performed in freshly isolated human PBMCs as a dose-finding and feasibility step to establish an optimal working concentration of 5 μM. CsA (Sandimmune; Novartis, Basel, Switzerland; 250 mg/mL, 41.6 mM stock) was serially diluted in 0.9% normal saline (NS) to generate a 416 μM intermediate stock. PBMCs (1.5 million cells per chamber) were suspended in MiR05 respiration medium and loaded into Oroboros O2k chambers following standard air calibration. After baseline oxygen consumption was recorded, 24 μL of the 416 μM intermediate stock was injected to achieve a final chamber concentration of 5 μM. Following a 5-min stabilization period, 5 mM DFP was administered to induce mitochondrial dysfunction and evaluate the extent of CsA-associated preservation of mitochondrial respiration. In this PBMC model, CsA at 5 μM was associated with preservation of mitochondrial respiration without evidence of cytotoxicity and was therefore selected to inform in vivo dosing.

For in vivo rodent studies, two CsA paradigms were evaluated: (1) post-exposure treatment and (2) pretreatment prior to DFP exposure. In the post-exposure treatment group, a continuous intravenous infusion of CsA was administered at 5 mg/kg over one hour immediately following DFP exposure. In the pretreatment group, CsA was administered one hour prior to DFP exposure to allow adequate systemic distribution and peak pharmacologic activity during the anticipated period of mitochondrial permeability transition pore (mPTP) activation. Prior studies have demonstrated that CsA-mediated cyclophilin D inhibition and mPTP modulation occur rapidly following systemic administration and are commonly evaluated within 30–120 min after dosing in experimental mitochondrial injury models.

The CsA infusion dose was prepared by a 1:250 dilution from stock, yielding a final concentration of 1 mg/mL for precise intravenous delivery. A CsA-only control group was not included in the rodent experiments in this preliminary feasibility study. However, CsA-only exposure was evaluated separately in PBMC experiments and did not demonstrate independent mitochondrial effects. The dosing strategy was informed by prior laboratory experience using CsA in experimental traumatic brain injury models targeting mitochondrial dysfunction and mPTP modulation [9, 10].

### Tissue and Whole Blood Extraction and Preparation

Upon completion of the protocol described above, all animals were euthanized, after which brain tissue and whole blood were immediately obtained. Approximately 4 mL of whole blood was obtained through cardiac puncture for plasma. PBMC experiments described in this study were performed separately using blood obtained from healthy human donors, as described above; PBMCs were not isolated from the experimental animals. Using Ficoll-Paque™ PLUS (GE Healthcare), a 50 mL Leucosep tube (Greiner Bio-One), and centrifugation, a population of PBMCs was isolated from the buffy coat.

Brain tissue immediately underwent rapid but gentle dissection and was maintained on ice throughout processing. Dissected cortical tissue was transferred into ice-cold isolation buffer and carefully cleaned of visible blood vessels and connective tissue. Tissue samples were weighed and prepared as homogenates in MiR05 respiration buffer to achieve an approximate concentration of 100 mg tissue/mL buffer. Tissue was manually minced and homogenized using a Potter homogenizer with slow spiral strokes while minimizing heat generation. Homogenates were maintained on ice and protected from light exposure until use in the Oroboros O2k chambers.

For mitochondrial respiration experiments, approximately 22 µL of tissue homogenate was added directly into MiR05-containing chambers and analyzed using high-resolution respirometry with a standardized substrate–uncoupler–inhibitor titration (SUIT) protocol. The protocol included sequential administration of digitonin, malate, pyruvate, ADP, glutamate, succinate, oligomycin, FCCP, rotenone, antimycin A, ascorbate/TMPD, and sodium azide to evaluate multiple oxidative phosphorylation (OXPHOS) and electron transport system (ETS)-linked respiratory states. Time from tissue acquisition to mitochondrial respirometry was approximately 2.5 h, consistent with established tissue homogenate viability protocols [11, 12].

### Measurement of Mitochondrial Respiration

Mitochondrial respiratory function was assessed using high-resolution respirometry with an Oroboros Instruments Oroboros O2k-FluoRespirometer according to a substrate–uncoupler–inhibitor titration (SUIT) protocol adapted from our prior work. Respiration was evaluated in rodent brain homogenate as well as human peripheral blood mononuclear cells (PBMCs). All experiments were performed in MiR05 respiration buffer at 37 °C with continuous stirring, and oxygen flux was recorded using DatLab 7 software (Oroboros Instruments). For human PBMC experiments, cells were permeabilized with digitonin (1 μL per 1 × 10^6^ cells) to permit substrate access to the mitochondria normalized to cell count (per 1 × 106 cells).

The SUIT protocol sequentially evaluated multiple respiratory states across the electron transport system. LEAK respiration supported by pyruvate and malate (LEAK_PM_) was first established following the addition of pyruvate (5 mM) and malate (5 mM) in the absence of adenosine diphosphate (ADP). Oxidative phosphorylation supported by pyruvate and malate (OXPHOS_PM_) was then stimulated by the addition of ADP (1 mM). Subsequent addition of glutamate (5 mM) generated maximal Complex I-supported oxidative phosphorylation (OXPHOS_CI_). Convergent Complex I and Complex II oxidative phosphorylation capacity (OXPHOS_CI+CII_) was assessed following addition of succinate (10 mM).

To evaluate proton leak-associated respiration, oligomycin was added to inhibit ATP synthase and generate oligomycin-induced LEAK respiration (LEAK_Omy_). Maximal uncoupled electron transport system capacity through convergent Complex I and II input (ETS_CI+CII_) was then measured by stepwise titration of the protonophore carbonyl cyanide p-(trifluoromethoxy) phenylhydrazone (FCCP). Rotenone was subsequently added to inhibit Complex I and isolate Complex II-supported uncoupled respiration (ETS_CII_).

Residual non-mitochondrial oxygen consumption was determined following inhibition of Complex III with antimycin A, and these values were subtracted from all respiratory states prior to analysis. Complex IV (CIV)-linked respiration was measured using N,N,N′,N′-tetramethyl-p-phenylenediamine (TMPD) and ascorbate as electron donors, followed by sodium azide inhibition to determine and subtract the chemical background oxygen flux. All respirometry data were acquired using DatLab 7 (Oroboros Instruments, Innsbruck, Austria) [13–16].

### Statistics and Data Analysis

Results for physiological variables, blood gases, and mitochondrial respiration are reported descriptively given the exploratory feasibility nature of the study and the small sample sizes. No formal statistical hypothesis testing was performed, and the data are presented to illustrate observed physiologic and respiratory response patterns across experimental groups. Summary data are presented as individual data points and, where appropriate, median with interquartile range (IQR). All data visualization and descriptive analyses were performed using GraphPad Prism (version 10.4.1, GraphPad Software, San Diego, CA, USA).

#### Result

### Baseline Characteristics, Blood Gas Chemistry, and Hemodynamic Variables

**Fig. 1:**
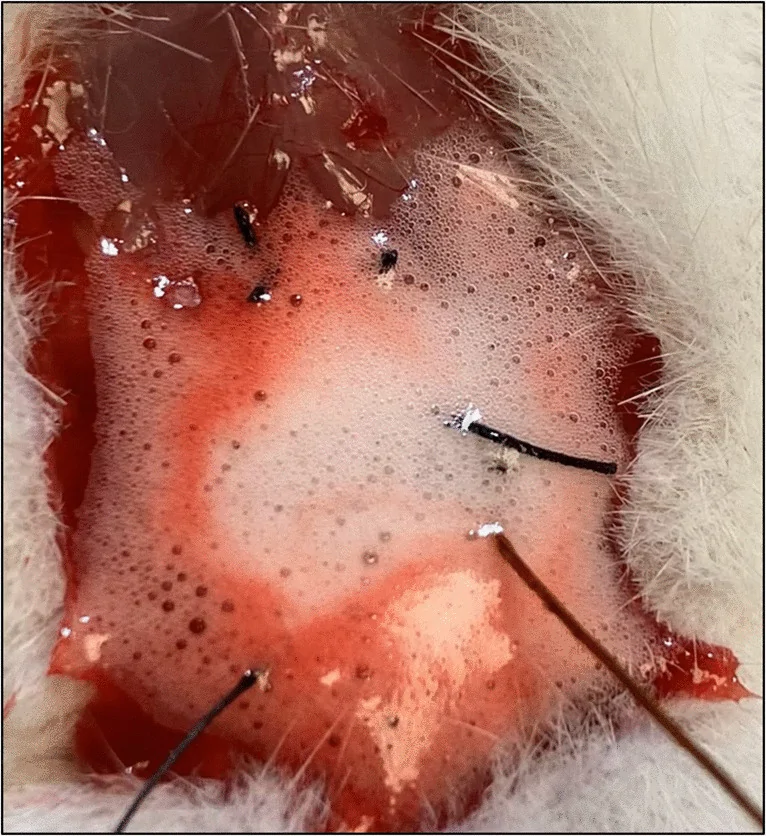
Representative rodent from the DFP exposure group demonstrating marked postmortem pulmonary edema of the trachea. During DFP exposure, peak inspiratory pressure (PiP) increased substantially, consistent with reduced pulmonary compliance and likely contributing to earlier mortality.

Baseline physiological parameters, including body weight, vital signs, and venous blood gas measurements obtained at the start of the experiment, were comparable across all animals (data not shown). Animals in the Control, Post-DFP CsA, and Pre-DFP CsA groups remained physiologically stable throughout the one-hour experimental period. In contrast, one of the two animals in the DFP exposure group developed progressive physiologic instability and underwent euthanasia at 45 min following DFP exposure. Physiological, blood gas, and mitochondrial respiration data obtained prior to euthanasia were included in the descriptive analyses (Table 1). Gross examination following euthanasia demonstrated pulmonary edema in the affected DFP-exposed animal (Fig. 1). The remaining DFP-exposed animals survived until completion of the experimental period. Individual physiological measurements and venous blood gas values for each experimental group are presented in Table 1.

**Table 1:** Physiological metrics and venous blood gas measurements are presented at the end of the one-hour experimental exposure period (baseline measurements were similar between groups and are not shown). The Control group did not receive any intervention, the DFP group received diisopropyl fluorophosphate (DFP) exposure alone, the Post-DFP CsA group received DFP exposure followed by cyclosporine A (CsA) infusion during the experimental period, and the Pre-DFP CsA group received CsA pretreatment one hour prior to DFP exposure. Individual animal values are presented due to the exploratory sample size and preliminary nature of the study. DFP = diisopropyl fluorophosphate; CsA = cyclosporine A.

Physiological metrics	Control (n = 2)	DFP (n = 2)	Post-DFP CsA (n = 2)	Pre-DFP CsA (n = 3)
Heart rate (BPM)	333, 341	420, 401	320, 350	306, 311, 301
Systolic (mmHg)	93, 98	210, 201	91, 110	104, 112, 104
Diastolic (mmHg)	63, 61	130, 110	61, 74	75, 69, 73
MAP (mmHg)	74, 73	157, 140	71, 86	85, 83, 83
Blood gas metrics	Control (n = 2)	DFP (n = 2)	Post-DFP CsA (n = 2)	Pre-DFP CsA (n = 3)
pH	7.41, 7.39	7.12, 7.20	7.29, 7.25	7.39, 7.39, 7.38
PCO₂ (mmHg)	39, 40	56, 55	52, 48	49, 49, 39
PO₂ (mmHg)	41, 38	42, 40	41, 41	38, 38, 38
HCO₃ (mmol/L)	25, 26	18, 16	21, 19	30, 30, 25
Lactate (mmol/L)	1.0, 0.9	4.1, 4.3	2.3, 2.5	0.70, 0.86, 0.97

## Mitochondrial Respiration

**Fig. 2:**
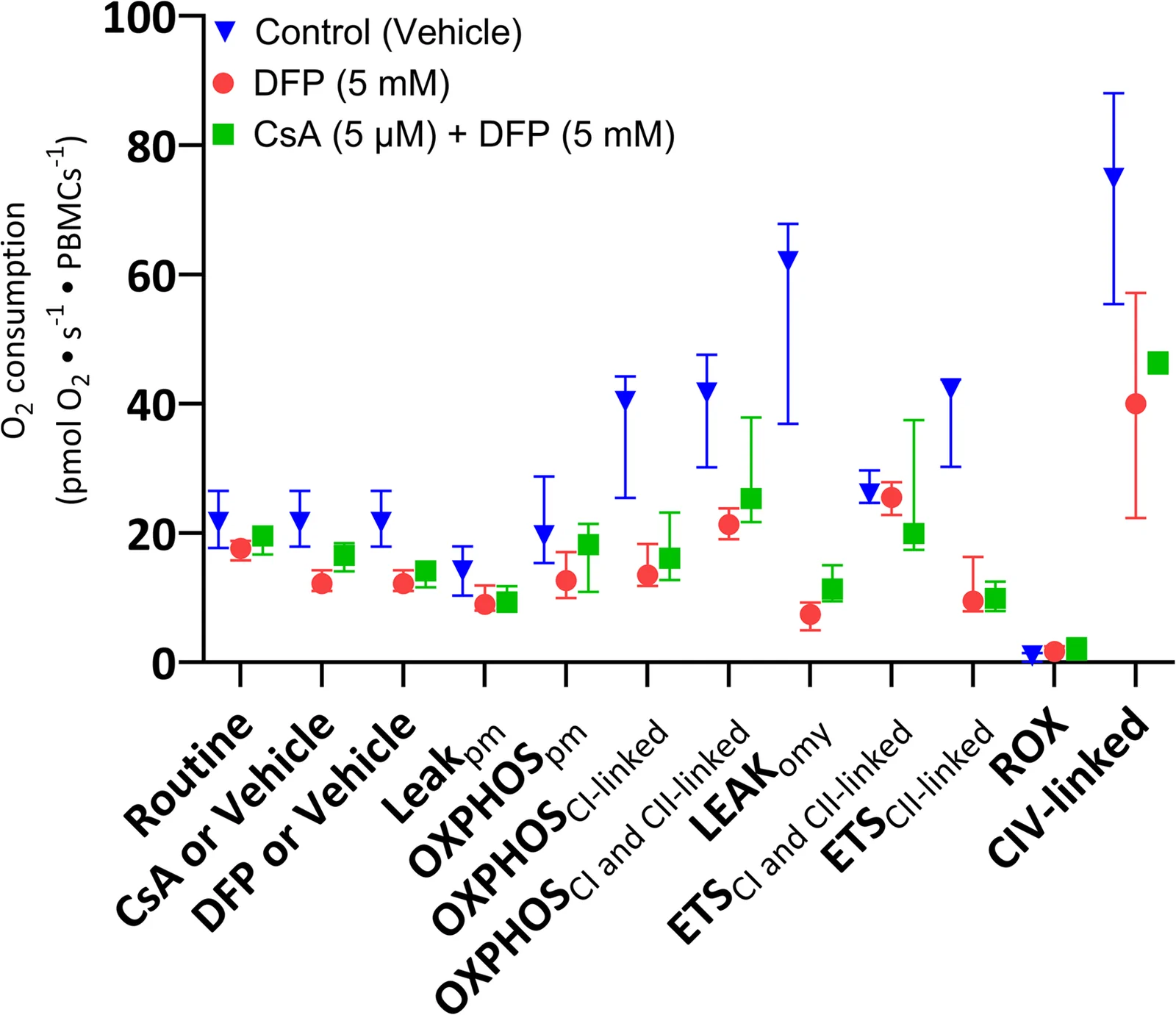
Preliminary PBMC mitochondrial respiration measurements across multiple respiratory states following DFP exposure and CsA pretreatment. Human donor-derived peripheral blood mononuclear cells (PBMCs) were exposed to DFP (5 mM) alone or pretreated with cyclosporine A (CsA; 5 uM) prior to DFP exposure. Across multiple respiratory states, DFP exposure was descriptively associated with lower mitochondrial oxygen consumption relative to contro PBMCs, while CsA pretreatment was associated with relatively preserved respiration in select OXPHOS- and ETS-linked respiratory states. Data are presented descriptively as median with interquartile range due to the exploratory sample size and preliminary nature of the study. OXPHOS = oxidative phosphorylation; ETS = electron transport system; CIV = Complex IV-linked respiration

Mitochondrial oxygen consumption was assessed ex vivo in human donor-derived PBMCs and rodent cortical brain homogenates. Human donor-derived PBMCs were used in a separate set of experiments to establish DFP and CsA dosing using a pretreatment paradigm. In these PBMC experiments, conducted with 5–6 replicates per condition, DFP exposure was descriptively associated with reduced mitochondrial respiration across multiple oxidative phosphorylation (OXPHOS)- and electron transport system (ETS)-linked respiratory states. CsA pretreatment was associated with relatively preserved oxygen consumption in select respiratory states compared with DFP exposure alone (Fig. 2).

**Fig. 3:**
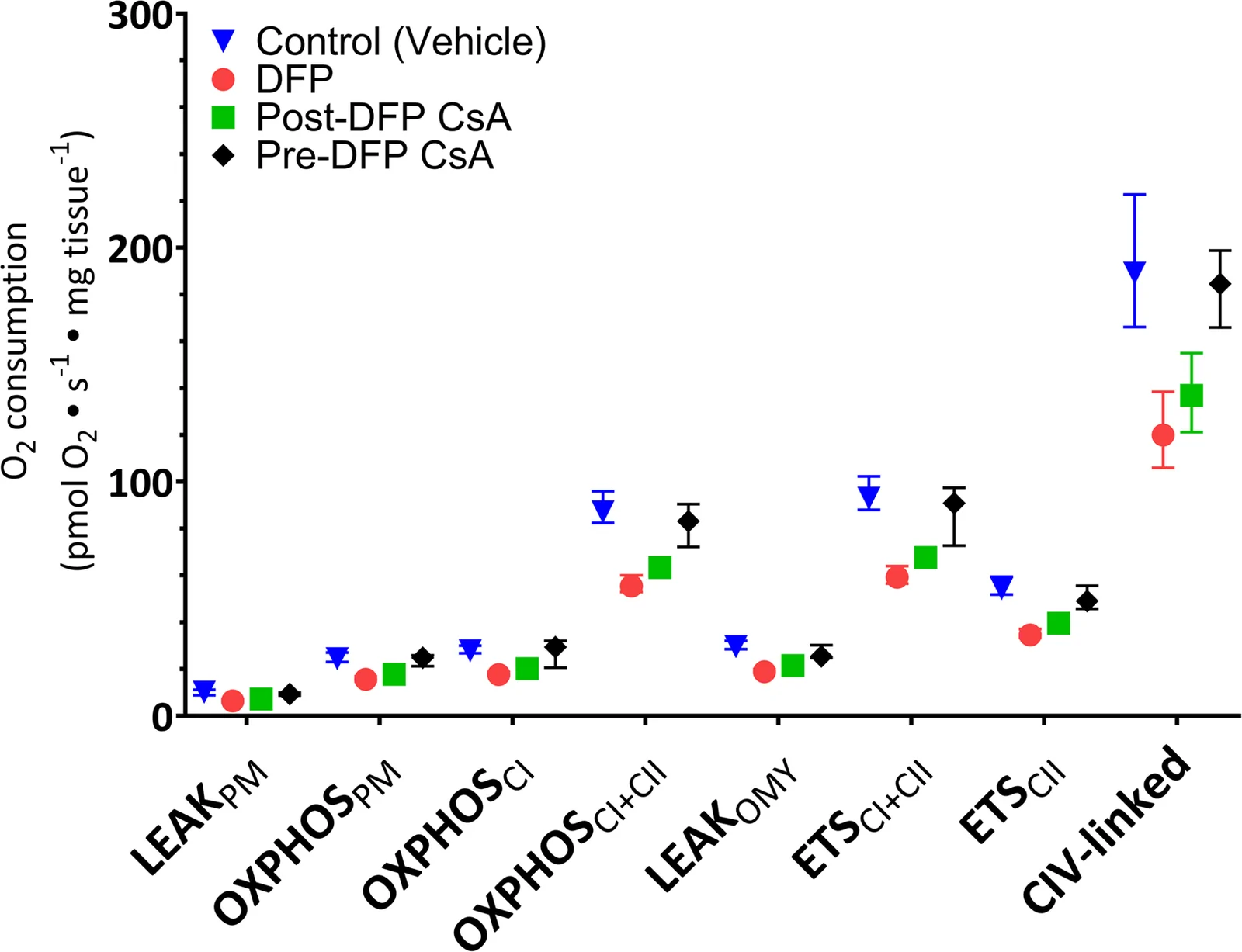
Preliminary rodent mitochondrial respiration measurements across multiple respiratory states following DFP exposure and CsA treatment paradigms. Animals were assigned to one of four groups: Control (Vehicle), DFP (exposure alone), Post-DFP CsA treatment, or Pre-DFP CsA pretreatment administered one hour prior to DFP exposure. Across multiple respiratory states, DFP exposure was descriptively associated with reduced mitochondrial oxygen consumption relative to controls. Post-DFP CsA treatment was associated with partial preservation of respiration across several respiratory states, while the Pre-DFP CsA group demonstrated respiratory profiles more closely approximating control values in select OXPHOS- and ETS-linked states. Data are presented descriptively as median with interquartile range due to the exploratory sample size and preliminary nature of the study. OXPHOS = oxidative phosphorylation; ETS = electron transport system; CIV = Complex IV-linked respiration

 In rodent experiments, mitochondrial respiration in cortical brain homogenates was markedly impaired following DFP exposure. Reductions were observed across multiple OXPHOS_CI+CII_-, ETS_CI+CII_-, and CIV respiratory states. Post-DFP CsA administration was associated with partial preservation of respiration across several respiratory states relative to DFP exposure alone. In addition, the Pre-DFP CsA group demonstrated respiratory profiles more closely approximating control values across select OXPHOS- and ETS-linked respiratory states (Fig. 3).

## Discussion

In this preliminary study, we utilized both cell-based and small-animal approaches to explore mitochondrial-targeted strategies in acute DFP toxicity. Initial experiments were conducted using human donor-derived PBMCs to establish preliminary DFP and CsA exposure conditions based on our prior experience with mitochondrial respiration assays in toxicologic models. A CsA pretreatment paradigm was evaluated in PBMCs and subsequently used to inform dosing strategies in the rodent model.

Across multiple respiratory states, DFP exposure was descriptively associated with lower mitochondrial oxygen consumption compared with control PBMCs. In contrast, CsA-pretreated PBMCs demonstrated relatively preserved mitochondrial respiration in several respiratory states, including OXPHOS- and ETS-linked conditions, although these findings were not uniform across all respiratory parameters. Given the exploratory nature of the study and limited sample size, these findings are presented descriptively and should be interpreted as preliminary feasibility observations rather than evidence of therapeutic efficacy.

Using dosing parameters informed by the human PBMC experiments, we next leveraged our established rodent platform for acute poisoning, which has previously been applied to toxicologic models including carbon monoxide and cyanide exposure [11, 12, 17, 18]. In this feasibility study, we developed an acute DFP poisoning model incorporating CsA administration informed by our PBMC data. Although the rodent sample size was limited (n = 2 per group), clear physiological and mitochondrial perturbations were observed following DFP exposure, which were partially attenuated in rodents treated with CsA.

Compared with control animals, rodents exposed to DFP demonstrated marked alterations in cardiovascular and respiratory physiology, including elevated heart rate and blood pressure, accompanied by increased peak inspiratory pressures and gross findings of pulmonary edema in one animal (Fig. 1).These changes preceded progressive physiologic instability in one DFP-exposed animal, which underwent euthanasia 45 min following exposure. Additional findings included diarrhea and combined metabolic and respiratory acidosis. In contrast, rodents treated with CsA following DFP exposure demonstrated relatively preserved physiologic parameters with less severe metabolic derangements on blood gas analysis. Mild diarrhea was observed, and minimal pulmonary secretions were noted on postmortem examination.

CsA is a cyclic peptide best known for its immunosuppressive effects mediated through calcineurin inhibition; however, its ability to inhibit opening of the mPTP has emerged as a key mechanism of interest in models of mitochondrial injury [9, 10]. By limiting mPTP opening, CsA has been associated with preservation of mitochondrial membrane potential, reduced calcium influx, and reduced release of pro-apoptotic factors in prior experimental models of mitochondrial injury. Given that DFP exposure has been associated with mitochondrial dysfunction and oxidative stress in prior experimental studies, the relatively preserved mitochondrial respiration observed in CsA-exposed rodents may support further investigation of mitochondrial-targeted strategies in acute organophosphate toxicity.

Several limitations warrant consideration. Foremost, this study was exploratory and limited by small sample sizes, precluding formal statistical inference. In addition, while rodent physiology does not fully reproduce human responses, rodent models remain well established in OP research and provide a practical platform for mechanistic and therapeutic investigation. The timing of the CsA paradigms evaluated in this study also represents an important limitation, as both pretreatment and immediate post-exposure administration strategies do not allow assessment of reversal of established organophosphate-induced injury or delayed-treatment scenarios that may more closely reflect real-world exposures. Additionally, isoflurane anesthesia has previously been associated with effects on mitochondrial respiration, including modulation of Complex I activity and mPTP function. Although all experimental groups underwent identical anesthetic protocols, and control animals were included to account for potential anesthetic-associated effects, these interactions may still represent a potential confounding factor when interpreting mitochondrial respiratory findings. Despite these limitations, the combined use of complementary cell-based and rodent models allowed exploratory evaluation of mitochondrial respiratory changes across multiple experimental systems. Future studies incorporating larger sample sizes, survivorship models, and delayed-treatment paradigms will be necessary to further evaluate these preliminary findings. These preliminary findings support the feasibility of integrating mitochondrial respiratory phenotyping into translational models of organophosphate toxicity and may inform future investigation of mitochondrial-targeted countermeasure strategies.
